# Acute Pancreatitis Caused By Mushroom Poisoning: A Report of Two Cases

**DOI:** 10.1177/2324709615627474

**Published:** 2016-01-21

**Authors:** Samet Karahan, Abdulsamet Erden, Ali Cetinkaya, Deniz Avci, Adile Irfan Ortakoyluoglu, Hatice Karagoz, Kadir Bulut, Mustafa Basak

**Affiliations:** 1 Akdeniz University School of Medicine, Antalya, Turkey; 2 Hacettepe University School of Medicine, Ankara, Turkey; 3 Kayseri Training and Research Hospital, Kayseri, Turkey; 4 Acibadem Hospital, Kayseri, Turkey

**Keywords:** acute pancreatitis, mushroom, poisoning, *Amanita phalloides*, *Lactarius volemus*

## Abstract

Of the more than 5000 species of mushrooms known, 100 types are toxic and approximately 10% of these toxic types can cause fatal toxicity. A type of mushroom called *Amanita phalloides* is responsible for 95% of toxic mushroom poisonings. In this article, we report 2 cases of mushroom poisonings caused by *Lactarius volemus*, known as *Tirmit* by the local people. The patient and his wife were admitted to the emergency room with abdominal pain, nausea, and vomiting 20 hours after consuming *Lactarius volemus*, an edible type of mushroom. The patients reported that they had been collecting this mushroom from the mountains and eating them for several years but had never developed any clinicopathology to date. Further examination of the patients revealed a very rare case of acute pancreatitis due to mushroom intoxication. The male patient was admitted to the intensive care unit while his wife was followed in the internal medicine service, because of her relative mild clinical symptoms. Both patients recovered without sequelae and were discharged. In this article, we aimed to emphasize that gastrointestinal symptoms are often observed in mushroom intoxications and can be confused with acute pancreatitis, thus leading to misdiagnosis of patients. Early diagnosis and appropriate treatment can improve patients’ prognosis and prevent the development of complications.

## Introduction

**Figure 1.**
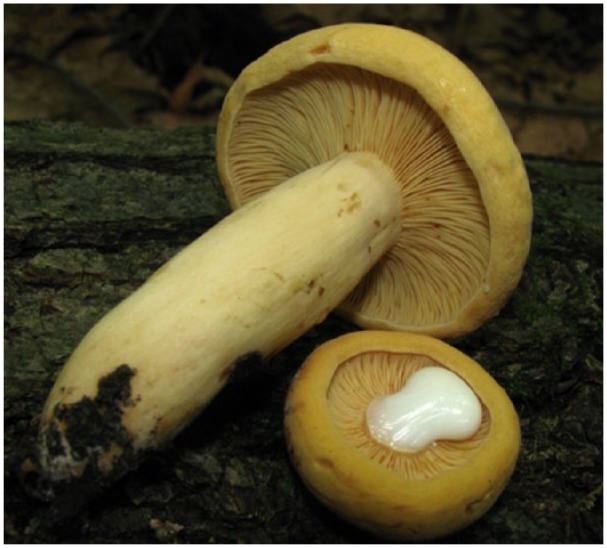
*Lactarius volemus* (popularly known as *Tirmit* in Central Anatolia).

In Turkey, about 2400 mushroom species are known, 100 types of which are reported to be toxic and 10 types fatal.^1^ Of the toxic types, *Amanita phalloides*, a very toxic and dangerous mushroom, is the most famous and is responsible for approximately 95% of fatal mushroom poisonings in Turkey^2^ There are different organ dysfunctions when an individual has a mushroom poisoning. But acute pancreatitis is a rare complication of mushroom poisoning. Acute pancreatitis and mushroom poisoning’s early symptoms are similar. Early recognition and appropriate therapy for acute pancreatitis and mushroom poisoning may improve prognosis and complications. In this article, we report 2 cases—a married couple—of acute pancreatitis admitted to the emergency room with abdominal pain, nausea, and vomiting after consuming *Lactarius volemus* (*Tirmit*; Figure 1), which is an edible type of mushroom.

## Case 1

**Figure 2.**
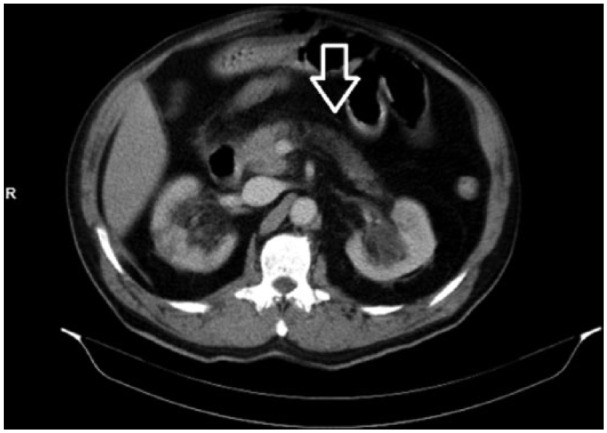
Axial computed tomography image of Case 1. Loss of lobulation of the pancreas and small amounts of peripancreatic fluid are observed.

A 73-year-old male patient was admitted to the emergency room with abdominal pain, nausea, and vomiting. He had a history of diabetes mellitus type 2, hypertension, and coronary artery disease. He was taking ramipril 10 mg/day, metoprolol 50 mg/day, acetylsalicylic acid 100 mg/day, and premixed insulin (twice a day). He did not consume alcohol. He reported ingesting a type of mushroom, known as *Tirmit* by the local people, 20 hours previously. He also reported that he knew this type very well and had been eating it for years although it had never caused any problems before. On admission, he was awake, fully oriented, and cooperative. His vital signs were as follows: blood pressure, 150/90 mm Hg; heart rate, 110/minute; temperature, 36.7°C. He had hypo-active bowel sounds. There was diffuse tenderness and defense on epigastric and periumbilical sides but no rebound. His initial laboratory tests were as follows: white blood cells (WBC), 21 600; neutrophils, 17 100; hemoglobin, 13.2 g/dL; platelets, 339 000/mm^3^; glucose, 522 mg/dL; creatinine (Cr), 2.1 mg/dL; aspartate aminotransferase (AST), 23 U/dL; alanine aminotransferase (ALT), 21 U/dL; sodium, 129 mEq/L; potassium, 3.3 mEq/L; amylase, 1148 U/L; and lipase, 2204 U/L. Other biochemical parameters were normal. His arterial blood gas values were as follows: pH 7.37; partial pressure of carbon dioxide, 36 mm Hg; and bicarbonate, 19.8 mmol/dL. Urine test was positive for glucose and negative for ketone. Abdominal computed tomography demonstrated the loss of pancreatic contour lobulation and a small amount of peripancreatic liquid (Figure 2). The patient’s oral intake was stopped and nasogastric decompression was performed. His laboratory findings on the second day following insulin and potassium infusion, analgesic treatment, and rehydration with liquids were as follows: WBC, 12 100; neutrophils, 11 100; Cr, 1.8 mg/dL; and amylase, 2025 U/L. He did not have any complaints of pain. On follow-up, amylase and Cr levels decreased progressively and returned to normal levels. The patient was discharged on the sixth day of treatment.

## Case 2

A 73-year-old female patient—the spouse of the male patient in Case 1—was admitted to the emergency room with complaints of nausea and vomiting after consuming the same mushroom. She did not have abdominal pain. On physical examination, her vital signs were normal and she had minimal tenderness on the upper abdomen. Her laboratory findings were as follows: WBC, 15 000; neutrophils, 14 100; amylase, 317 U/L; and lipase, 280 U/L. Other biochemical parameters and abdominal ultrasonography were normal. She was admitted to the internal medicine service because of her relative mild clinical symptoms. The patient’s oral intake was stopped and fluid replacement therapy was performed. On follow-up, the clinical symptoms disappeared and amylase values returned to normal levels on the second day. She was discharged on the third day of treatment.

## Discussion

Mushrooms are parts of fungi, completely different from animals and plants. Edible mushrooms are one of the important foodstuffs for people living in rural areas, but they can sometimes be dangerous or even cause death as some are very poisonous. The problem is that poisonous and nonpoisonous types cannot be easily distinguished every time. *Amanita phalloides* is the most dangerous of these mushrooms; it may even cause acute liver failure requiring liver transplantation.^3^ Although the clinical findings vary depending on the degree of toxicity and the clinical formation rate, patients may have insignificant clinical findings. The clinical spectrum may range from nonspecific gastroenteritis to acute fulminant liver failure. In fact, the major point of the treatment is to prevent the toxicity by distinguishing edible, nonpoisonous mushrooms from other poisonous mushrooms. In addition, it should be noted that the toxins of many mushrooms cannot be removed by cooking, freezing, or preserving. The most reliable way is to avoid alcohol intake while eating mushrooms.^4^ There are no standard and antidote treatments defined for mushroom poisonings. The treatment consists of fluid and electrolytes replacement and gastric lavage and activated charcoal to prevent the absorption of toxins from the gastrointestinal system in the earlier hours. Benzylpenicillin (penicillin G) and silibinin/silymarin are proven effective antidotes but they may not be beneficial in the case of fulminant hepatitis. Although the efficiency of drugs has not yet been proved, thioctacid acid, cimetidine, *N*-acetylcysteine, and corticosteroids are also used in the treatment of mushroom poisonings. Vitamin K may be required in the case of coagulopathy.^5^ Hypoglycemia, prolonged prothrombin time, and significant increases in bilirubin and transaminase levels are the poor prognostic factors for liver failure.^6^ Renal failure is related with hepatorenal syndrome, and the direct toxic effect of α-amanitin on kidneys.^2^ Renal failure in our case was probably due to prerenal azotemia secondary to hypovolemia, which occurred after nausea and vomiting, and was resolved by fluid replacement. Prolonged prothrombin time, increases in liver function tests, and increases in blood ammonia levels with grade 2 to 3 hepatic encephalopathy or international normalized ratio level above 6 without encephalopathy are signs of fulminant liver failure and indications for liver transplantation.^7^ Acute pancreatitis is defined as the presence of acute abdominal pain clinically with increases of pancreatic enzymes in serum and/or urine, and the radiological changes in the pancreas.^8^ There was acute pancreatitis caused by mushrooms in our patient although he was an experienced hunter.

In the literature, there are 3 mushroom intoxication cases of fulminant liver failure in addition to acute pancreatitis,^3^ and also a number of cases of acute pancreatitis without liver failure reported in Turkey.^9^ The patient had a history of eating the same type of mushroom many times before so the question “Why he developed acute pancreatitis this time” could not be explained. The mechanism that causes acute pancreatitis in mushroom poisonings has not been explained for rare cases in the literature either, although the probable mechanism is thought to be the targeting of mushroom toxins directly on pancreas B cells. Additionally, it is believed that hypoglycemia occurs after the release and increases in insulin and c-peptid levels, due to the direct toxic effect on the pancreas.^10^ We did not observe hypoglycemia or any changes in bleeding parameters, and AST/ALT levels decreased gradually in our cases. Because the patients knew this type of mushroom and had been eating it many times for years, we believe that there may be a different mechanism or mechanisms causing acute pancreatitis. But it seems that, the first case was more serious case than the second one, which may be due to the use of ramipril, which is known as a cause of acute pancreatitis. It may be additive effects of mushroom poisoning and ramipril usage as a cause of acute pancreatitis. Angiotensin-converting enzyme inhibitors–associated pancreatitis is thought to reflect localized angioedema of the pancreas^11^. It is not studied whether *Lactarius volemus–*like mushrooms cause or deepen the pancreatic edema.

## Conclusion

It must be remembered that mushroom toxicity can lead to serious organ failures—especially liver and renal failures—and even death. Furthermore, gastrointestinal symptoms are often observed in mushroom intoxications and can be confused with acute pancreatitis, thus patients may be misdiagnosed. If acute pancreatitis is present, appropriate treatment strategies should be performed for pancreatitis as well as mushroom intoxication.
